# Epidemiology of Early Monkeypox Virus Transmission in Sexual Networks of Gay and Bisexual Men, England, 2022

**DOI:** 10.3201/eid2810.220960

**Published:** 2022-10

**Authors:** Amoolya Vusirikala, Hannah Charles, Sooria Balasegaram, Neil Macdonald, Deepti Kumar, Ceri Barker-Burnside, Kerry Cumiskey, Michelle Dickinson, Michelle Watson, Oluwakemi Olufon, Katie Thorley, Paula Blomquist, Charlotte Anderson, Thomas Ma, Hamish Mohammed, Samantha Perkins, Karthik Paranthaman, Petra Manley, Obaghe Edeghere, Katy Sinka, Mateo Prochazka

**Affiliations:** UK Health Security Agency, London, UK

**Keywords:** monkeypox, viruses, epidemiology, gay and bisexual men, MSM, transmission, England, United Kingdom, men who have sex with men

## Abstract

After community transmission of monkeypox virus was identified in Europe, interviews of 45 case-patients from England indicated transmission in international sexual networks of gay and bisexual men since April 2022. Interventions targeting sex-on-premises venues, geospatial dating applications, and sexual health services are likely to be critical for outbreak control.

Monkeypox is a zoonotic viral infection endemic to West and Central Africa. Human-to-human transmission of monkeypox virus (MPXV) is thought to be primarily through close skin-to-skin contact; other transmission routes include respiratory secretions and fomites (*1*). MPXV incubation period is 5–21 days (*1*). Before 2022, sporadic cases reported in England had been directly linked to travel from monkeypox-endemic areas or were identified as household or healthcare contacts of case-patients (*2*–*4*). However, in May 2022, several countries in Europe reported sustained human-to-human transmission of MPXV, primarily in sexual networks of gay, bisexual, and other men who have sex with men (GBMSM) (*5*–*7*).

By May 25, 2022, in England, 85 PCR-confirmed monkeypox cases were reported, 82 with known or suspected links to transmission in GBMSM sexual networks (*7*). Sexual networks are social networks in which persons are connected by sexual activity. An urgent need for information about the epidemiology of this unusual pattern of MPXV transmission led the UK Health Security Agency (UKHSA) to deploy rapid sexual health questionnaires to persons with confirmed MPXV infection. We analyzed the findings and implications for public health action.

This study was undertaken for health protection purposes under permissions granted to UKHSA to collect and process confidential patient data under Regulation 3 of The Health Service (Control of Patient Information) Regulations 2020 and Section 251 of the National Health Service Act 2006. All data were anonymized during analysis, and records were stored securely.

## The Study

Persons with confirmed MPXV infection were identified by PCR at the Rare and Imported Pathogens Laboratory at UKHSA, according to standard procedure, and reported to local health protection teams, who were responsible for public health management. Case-patients with known or suspected links to transmission in GBMSM sexual networks were invited for follow-up interviews focused on their sexual health; phone interviews were conducted during May 25–30, 2022, and followed a structured questionnaire. Participation in the follow-up interviews was voluntary, and verbal consent was obtained after the context and rationale for the additional interview was explained. The questionnaire information was similar to that captured in previous outbreaks: demographics (sex, age, sexual orientation, ethnicity, country of birth); potential exposures in the 21 days before symptom onset (travel history, exposure events, sexual behavior); markers of sexual behavior associated with higher risk of acquiring sexually transmitted infections (STIs) (previous STI, number of sex partners in past 3 months); and HIV prevention and care (status, HIV preexposure prophylaxis [PrEP], HIV treatment) (*8*,*9*).

Sexual activity was defined as any direct contact of a sexual nature, including kissing, oral, and penetrative sex. Group sex was defined as sexual activity with >1 person at a time. Chemsex was defined as use of drugs such as GHB (gamma-hydroxybutyrate), crystal methamphetamine, or mephedrone during sex. Exposure sites/events included sex-on-premises venues (commercial venues where sexual activity occurs), private sex parties, cruising, and international events/festivals. Cruising was defined as sexual activity with anonymous partners in public outdoor spaces. Private sex parties were defined as group sex in a household, and when not explicitly mentioned, private sex parties were inferred if a person reported group sex and chemsex outside sex-on-premises venues.

We securely recorded data by using the online questionnaire tool Snap 11 Professional (https://www.snapsurverys.com) and extracted data for analysis on May 30, 2022. We used Stata 17.0 (https://www.stata.com) to clean and summarize the data to provide descriptive statistics. To assess participation bias, we used Mann-Whitney U, χ^2^, and Fisher exact tests to compare case-patients by age, region of residence, and travel history; data were sourced from the public health case management system (HPZone, https://hpzone.phe.gov.uk). To identify time when community transmission started and potential routes of importation, we plotted onset of first symptoms (prodrome or rash), travel, and venue attendance dates on timelines.

Of 82 case-patients with known or suspected links to transmission in GBMSM sexual networks identified up to May 25, 2022 (*7*), we re-interviewed 45 (55%) for this specific detailed questionnaire (Figure). Reasons for not interviewing included lack of contact details (n = 11), declining to participate (n = 4), and inability to re-establish phone contact (n = 22). We found no significant differences between interviewed and noninterviewed case-patients in terms of age, region of residence, or travel history (p>0.05).

**Figure:**
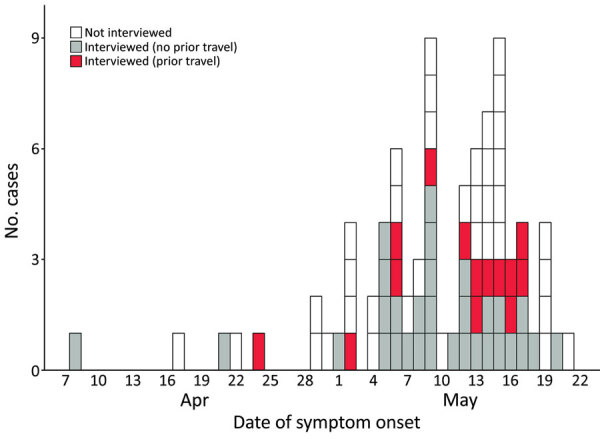
Epidemic curve of monkeypox cases, by symptom onset date and patient travel status within 21 days before symptom onset, England, 2022.

Symptom onset dates were April 8–May 20, 2022. All cases were diagnosed in May; because of suspected community transmission, some persons with ulcerative and vesicular rashes were re-called for MPXV testing and retrospectively identified as early confirmed case-patients (*7*). Of 31 interviewed case-patients who did not travel abroad in the 21 days before symptom onset, symptom onset began in April 2022, including for 1 case-patient who subsequently traveled to an international event. Throughout May 2022, symptoms developed in several case-patients who returned to the United Kingdom after traveling in Europe (Figure).

Nearly all interviewed case-patients self-identified as either gay or bisexual (98%, 44/45). HIV status was reported by 43 case-patients: 74% (32/43) were HIV negative, of which 91% (29/32) were receiving PrEP; 11/43 (26%) reported living with HIV and all were receiving HIV treatment. Although most (29/45, 64%) case-patients reported attending sex-on-premises venues, festivals, private sex parties, or cruising grounds in the 21 days before symptom onset, 16/45 (36%) did not report such exposures (Table). Of those, 12/16 (75%) reported sexual activity with new partners; 10/12 (83%) met via geospatial dating applications.

**Table:** Characteristics and exposures of 45 interviewed persons with confirmed monkeypox, England, 2022*

Variables	No. (%)
Cisgender men	45 (100)
Ethnicity	
White	35 (78)
Black	1 (2)
Asian	3 (7)
Mixed	3 (7)
Other	3 (7)
Place of birth	
United Kingdom	19 (43)
Europe, not including United Kingdom	12 (27)
South America	5 (11)
Other	8 (18)
Unknown	1 (2)
Region of residence	
London	39 (87)
Other regions in the United Kingdom	6 (13)
Sexual orientation	
Gay	40 (89)
Bisexual	4 (9)
Heterosexual	0
Other	1 (2)
No. sexual contacts in past 3 mo	
1	4 (9)
2–3	6 (14)
4–9	13 (30)
>10	20 (47)
Prefer not to say/unknown	2 (3)
HIV prevention and care	
HIV negative	32 (71)
Receiving PrEP	29 (91)
Living with diagnosed HIV	11 (24)
Receiving HIV treatment	11 (100)
Undetectable viral load	10 (91)
Prefer not to say/unknown HIV status	2 (4)
History of STI in past year	
Yes	27 (60)
No	18 (40)
Travel abroad within 21 d before symptom onset	
Yes, reported sexual activity	9 (20)
Yes, but no sexual activity	5 (11)
No	31 (69)
Exposure events within 21 d before symptom onset†	
Festivals outside of the United Kingdom	5 (11)
Sex-on-premises venues	20 (44)
Private sex parties	9 (20)
Cruising grounds	7 (16)
None of the above	16 (36)
Sexual activity within 21 d before symptom onset‡	
Sexual activity with new partners	37 (82)
Sexual activity with one-time partners	34 (76)
Sexual activity with occasional partners	24 (53)
Sexual activity with established partners	12 (27)
Sexual activity with women	2 (5)
Group sexual activity	20 (44)
Chemsex	10 (22)
Sexual activity with partners who are not regular UK residents	11 (24)
Sexual activity in locations different from city/town of residence	13 (30)
Sexual activity with partners met via geospatial dating apps	28 (64)
No sexual activity reported	2 (4)

*Sexual activity was defined as direct contact of a sexual nature, such as kissing, oral sex, and penetrative (vaginal, anal) sex. Some categories in this table were collapsed to avoid deductive disclosure. Median (interquartile range) age, 40 (32–43) y. PrEP, pre-exposure prophylaxis; STI, sexually transmitted infection. †Sex-on-premises venues, private sex parties and cruising grounds are activities that took place either in the United Kingdom or abroad. ‡New partner, person with whom the index case-patient is likely to have had sex on >1 occasion; one-time partner, person with whom the index case-patient has had sex on 1 occasion only; occasional partner, person with whom the index case-patient has had sex on >1 occasion and with whom there is an expectation of sex again on a sporadic or regular basis; established partner, primary partner or secondary partner (e.g., a long-term affair) (*10*); chemsex, use of drugs such as GHB (gamma-hydroxybutyrate), crystal methamphetamine, or mephedrone during sex.

## Conclusions 

Our findings suggest that sustained domestic MPXV transmission in sexual networks of GBMSM in England has been occurring since at least April 2022, with potential importations and exportations from other countries in Europe. MPXV transmission in sexual networks has been suggested for outbreak investigations in Nigeria (*11*). The origin and prevalence of MPXV infection among GBMSM is unknown, but international dissemination was probably catalyzed by travel and resumption of events after lifting of restrictions associated with the COVID-19 pandemic.

Our findings show that domestic transmission seems to be sustained by sexual contact in dense sexual networks of GBMSM, often between multiple new partners who are probably difficult to contact trace because of one-time contacts. Similar to previous outbreaks of other STIs among GBMSM (*12*,*13*), contact tracing alone might not be effective as the primary control intervention to stop transmission.

Our findings also suggest that a substantial element of MPXV transmission in England occurs within sex-on-premises venues. To achieve outbreak control, targeted interventions for venues and their users are vital, including supporting enhanced cleaning of venues to prevent transmission via fomites, targeted health promotion to build awareness and inform risk management, and innovative approaches to support contact tracing of venue attendees (*14*). Designing and implementing these interventions requires community and stakeholder engagement.

Persons with a monkeypox diagnosis were in frequent contact with sexual health services and linked to care for HIV medication or PrEP. This link to existing services and programs provides an opportunity for policymakers to implement other interventions to reach those with highest need, including using smallpox Modified Vaccinia Ankara vaccine (Bavarian Nordic, https://www.bavarian-nordic.com) as PrEP for MPXV (*15*).

A substantial proportion of case-patients did not report specific exposure settings, stressing the need for wider interventions to reach all GBMSM in these sexual networks. The high use of geospatial dating applications highlights their utility as a platform for health promotion.

Limitations of this study include social desirability bias, participation bias, and evolving patterns of transmission, affecting their generalizability. Nonetheless, these analyses may provide information for the initial outbreak response in countries with similar transmission patterns. Because of the high interconnectivity of sexual networks, urgent multilateral action is needed to stop human-to-human transmission and avoid global endemicity.
